# Cancer‐testis antigen ACRBP expression and serum immunoreactivity in ovarian cancer: Its association with prognosis

**DOI:** 10.1002/iid3.534

**Published:** 2021-09-16

**Authors:** Lina Lin, Weixia Nong, Bin Luo, Yingying Ge, Xia Zeng, Feng Li, Rong Fan, Qingmei Zhang, Xiaoxun Xie

**Affiliations:** ^1^ Department of Histology and Embryology, School of Preclinical Medicine Guangxi Medical University Nanning China; ^2^ Central Laboratory, School of Preclinical Medicine Guangxi Medical University Nanning China; ^3^ Department of Histology and Embryology, School of Preclinical Medicine Guangxi Chinese Medicine University Nanning China

**Keywords:** ACRBP, cancer‑testis antigen, diagnostic marker, immunotherapy, ovarian cancer

## Abstract

**Introduction:**

Cancer testis (CT) antigens are attractive targets for cancer immunotherapy because of their expression restriction and immunogenicity. The acrosin binding protein (ACRBP) is a member of CT antigens. This study aimed to evaluate ACRBP expression and immunogenicity in ovarian cancer (OC).

**Methods:**

The expression level of ACRBP in OC tissues, normal ovarian tissues, and cell lines was detected via quantitative real‐time polymerase chain reaction (qRT‐PCR) and immunohistochemistry. We determined the levels of ACRBP antigen and antibody in serum samples collected from patients with OC and healthy donors using enzyme‐linked immunosorbent assays (ELISA), the level of ACRBP in cell‐cultured medium was also tested.

**Results:**

ACRBP mRNA and protein expressions were upregulated in OC tissues relative to normal tissue, especially highly expressed in epithelial ovarian cancer (EOC). Moreover, ACRBP expression was significantly correlated with International Federation of Gynecology and Obstetrics (FIGO) stage and chemosensitivity. Serological analysis showed that anti‐ACRBP antibody was detected in the sera of 16 of the 56 (28.5%) patients with OC but not in healthy donors. The area under the receiver operating characteristic curve for ACRBP antibody was 0.802 (95% confidence interval [CI]: 0.708–0.876), and the sensitivity and specificity for ACRBP antibody was 85.71% and 55.0%, respectively. Kaplan–Meier analysis revealed that the overall survival (OS) and disease‐free survival (DFS) in OC patients with high ACRBP expression were significantly lower than those with low expression (*p* = 0.040, *p* = 0.021). However, ACRBP antibody level was not associated with prognosis.

**Conclusion:**

ACRBP expression was upregulated in OC tissues and induced humoral immune response in patients with OC, suggesting that ACRBP is a potential prognostic biomarker and a target of tumor immunotherapy for OC.

## Graphical abstract

(1) Acrosin binding protein (ACRBP) was a restricted expression cancer‐testis antigen in cancers. Its expression was markedly upregulated in ovarian cancer tissues and induced humoral immune response in patients with ovarian cancer. Serological detection of ACRBP had high sensitivity in ovarian cancer. (2) The overexpression of ACRBP was associated with advanced stage of tumor, metastasis, and chemoresistance in ovarian cancer. High ACRBP expression was associated with a poor clinical outcome. (3) ACRBP also displayed inherent immunogenicity. Together with its restricted expression pattern and high specificity to cancer cells, ACRBP could be a potential target for prognostic evaluation and tumor‐specific antigen‐based immunotherapy for patients with ovarian cancer.

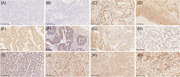

## 1 INTRODUCTION

Ovarian cancer (OC) is the most common and fatal gynecological malignancy and the fifth leading cause of cancer‐related deaths. In recent years, the incidence of OC has substantially increased among young women. OC mortality ranks first among malignant tumors of the female reproductive system. About 225,000 women worldwide are diagnosed with OC every year.^1^, ^2^ Among ovarian malignancies, epithelial ovarian cancer (EOC) is the most common pathological type, accounting for 85%–90% of OC cases. Given that the clinical symptoms of OC are hidden, most patients are already in the progressive stage when they are first diagnosed. Thus, OC is also known as the “silent killer” that seriously threatens women's health and life. Satisfactory cytoreductive surgery combined with postoperative platinum‐based chemotherapy is the standard treatment for OC. Despite advances in OC treatment, the 5‐year survival rate of patients with OC is hovering at about 30%–40%, and recurrence and metastasis rates remain severely high.^3^, ^4^ Nearly 90% of patients with Stage III OC recur within 5 years, and once OC has recurred and metastasized, the prognosis is extremely poor. Therefore, new therapeutic approaches for improving the survival of patients with OC must be developed. Various studies on relevant OC genes have provided a new notion for the targeted therapy and molecular diagnosis of OC. However, the lack of tumor specificity in some therapeutic targets may lead to on‐target adverse reactions. Hence, new targets for early diagnosis, prognosis, and therapies for OC must be explored.

Cancer‐testis (CT) antigens are a family of genes that are expressed in various types of human cancers, including OC. However, CT antigens are not expressed or restricted in normal tissues, except in the testis.^5^, ^6^ Owing to the restricted expression of CT antigens, and given that the testis is an immune‐exempt organ, the application of CT antigens to tumor immunotherapy will not cause autoimmune reactions. Therefore, CT antigens are regarded as ideal tumor‐specific immunotherapy targets.^7^ Substantial evidence supports the idea that CT antigens play important roles in tumor migration, invasion, and angiogenesis. In recent years, an increasing number of studies have focused on the identification of CT antigens. The family members of CT antigens reported thus far in OC include the MAGE family, NY‐ESO‐1, SSX, and CT45, which are categorized as CT‐X antigens. And BORIS, PRAME, PIWIL, and AKAP3/4, which are categorized as non‐X cancer/testis antigens (CTAs).^8^, ^9^, ^10^, ^11^

The acrosin binding protein (ACRBP), also called OY‐TES‐1 and CT23, is a member of the CT antigen family that was identified and named by Ono et al. in 2001. It is located on the short arm of chromosome 12. It has a total length of 9339 bp, containing 10 exons, and has a full‐length transcription unit of 1895 bp.^12^ Elevated ACRBP mRNA expression can be detected in various tumor tissues and cell lines, such as glioma, hepatocellular carcinoma, and colon cancer.^13^, ^14^, ^15^ In a previous study, we found that knockdown of ACRBP inhibits cell proliferation, prevents migration and invasion, arrests cell cycle, and promotes cell apoptosis of human mesenchymal stem cells (MSCs) and hepatocellular carcinoma cells.^16^, ^17^ Tammela et al.^18^ reported that 60% (60/100) of EOC tissues express the ACRBP, which was found to interact with the nuclear mitotic apparatus (NuMA) protein, suggesting that their interaction plays a role in paclitaxel resistance in EOC. Serological surveys detected antibodies against ACRBP in 3.5%–22.2% of patients with different types of cancer.^13^, ^14^, ^15^, ^18^ To date, no study has reported on the clinical importance of serum ACRBP levels in patients with OC. The value of ACRBP in OC diagnosis and prognosis remains unknown. Therefore, this topic is worthy of our further investigation.

In this study, we profiled the ACRBP expression in OC by using a public database and clinical samples. We performed a serological survey for ACRBP and confirmed its secretion in cultured OC cells. Furthermore, we evaluated the clinical importance of ACRBP expression and its serum antibody in patients with OC. We then investigated the relationship of ACRBP expression and ACRBP serum levels to the patients' overall survival (OS) and disease‐free survival (DFS).

## 2 MATERIALS AND METHODS

### 2.1 Bioinformatics analysis

To understand ACRBP expression in OC, we used the RNA sequence data obtained from the gene expression profiling interactive analysis (GEPIA) database (http://gepia.cancer-pku.cn/) to analyze differences in ACRBP expression between OC samples and normal ovarian tissue samples. The prognostic value of ACRBP was assessed using the Kaplan–Meier plotter website (http://kmplot.com/analysis/), which was utilized to assess the effects of 54k genes on survival in 21 cancer types, and the largest datasets included OC (*n* = 2190).

### 2.2 Cell lines and shRNA transfection

The OC cell lines SKOV3 and A2780, which are known to express ACRBP, were obtained from the Shanghai Cell Collection of the Chinese Academy of Science. The cell lines were cultured in RPMI‐1640 supplemented with 10% fetal bovine serum (FBS) at 37°C in an atmosphere containing 5% CO_2_. ACRBP shRNA lentiviral vector and negative control lentiviral vector were constructed by GenePharma. When the cells reached 60%–70% confluence, the vector was transfected into the SKOV3 and A2780 cells in accordance with the manufacturer's protocol. ACRBP mRNA expression was detected via quantitative real‐time polymerase chain reaction (qRT‐PCR). A conditioned medium harvested from OC cell cultures was prepared as follows: OC cells (10^6^/ml) were added into a 25 cm^2^ vented cap flask with 3 ml of RPMI‐1640 containing 10% FBS and incubated for 3 days. The cultured medium collected from each flask was centrifuged at 1000 rpm for 5 min and stored at −20°C until use.

### 2.3 Patients and specimens

Tissues and sera were collected from patients who underwent surgery at the Department of Obstetrics and Gynecology of the First Affiliated Hospital of Guangxi Medical University between November 2018 and January 2020. The tissues consisted of 65 OC tissues and 20 normal epithelial ovary tissues. Forty sera from healthy donors were obtained from routine physical examination. The pathological classification of OC was determined in accordance with the International Federation of Gynecology and Obstetrics (FIGO) system and the World Health Organization criteria. None of the patients received any neoadjuvant therapy, such as chemotherapy and radiotherapy before surgery. All specimens had been histologically and clinically diagnosed by two independent experienced pathologists. The clinical and pathological characteristics of the patients are given in Table 1. The research protocol was approved by the Ethical Review Committee of the First Affiliated Hospital of Guangxi Medical University (protocol No. 2018 [KY‐E‐124]). All patients were informed completely and signed informed consent. All experiments were conducted following the guidelines and regulations of the Ethical Review Committee of the First Affiliated Hospital of Guangxi Medical University and were conducted in accordance with the Declaration of Helsinki.

**Table 1:** Correlation between ACRBP expression and clinicopathological characteristics in OC tissues

Item		mRNA^a^	*n* (%)		Protein^b^	*n* (%)	
	No.	High	Low	*p*	High	Low	*p*
Age (years)							
<50	28	12 (42.8)	16 (57.2)	.267	15 (53.5)	13 (46.5)	.486
≥50	37	21 (56.8)	16 (43.2)		23 (62.2)	14 (37.8)	
FIGO stage							
I–II	30	10 (33.3)	20 (66.7)	.002	12 (40)	18 (60)	.005
III–IV	35	25 (71.4)	10 (28.6)		26 (74.3)	9 (25.7)	
Grade							
G3	36	20 (55.6)	16 (44.4)	.559	22 (61.1)	14 (38.9)	.629
G1 + G2	29	14 (48.3)	15 (51.7)		16 (55.2)	13 (44.8)	
Histopathology							
Serous	27	16 (59.3)	11 (40.7)	.993	19 (70.4)	8 (29.6)	.893
Mucious	14	8 (57.1)	6 (42.9)		9 (64.3)	5 (35.7)	
Endometriod	6	3 (50)	3 (50)		4 (66.7)	2 (33.3)	
Clear cell	5	3 (60)	2 (40)		3 (60.0)	2 (40.0)	
Others	13	8 (61.5)	5 (38.5)		7 (53.8)	6 (46.2)	
Serum CA125 (U/mL)							
<500	33	15 (45.5)	18 (54.5)	.058	16 (48.5)	17 (51.5)	.256
≥500	32	22 (68.8)	10 (31.2)		20 (62.5)	12 (37.5)	
Lymph node metastasis							
Positive	25	14 (56)	11 (44)	.208	16 (64)	9 (36)	.136
Negative	40	16 (40)	24 (60)		18 (45)	22 (55)	
Chemosensitivity							
Sensitive	45	20 (44.4)	25 (55.6)	.247	18 (40)	27 (60)	.026
Resistant	20	12 (60)	8 (40)		14 (70)	6 (30)	
Ki‐67							
<10%	13	6 (46.2)	7 (53.8)	.454	5 (38.5)	8 (61.5)	.133
≥10%	52	30 (57.7)	22 (42.3)		32 (61.5)	20 (38.5)	

Abbreviations: FIGO, International Federation of Gynecology and Obstetrics; OC, ovarian cancer.

^a^ High expression represents mRNA relative expression ≥3.2，low expression represents mRNA relative expression <3.2.

^b^ High expression represents + +/+++, low expression represents −/+.

### 2.4 qRT‐PCR analysis

Total RNA was extracted from OC tissues, normal ovarian tissues, and OC cells by using a Fast Pure Total RNA Isolation Kit (Vazyme Biotech Co., Ltd.) following the manufacturer's protocol. cDNA was then synthesized using a Revert AidTM First‐Strand cDNA Synthesis Kit (MBI Fermentas). qRT‐PCR was performed using SYBR Green PCR Master Mix (Applied Biosystems) following the manufacturer's instructions. Glyceraldehyde 3‐phosphate dehydrogenase (GAPDH) was used as an internal control. ACRBP mRNA level was analyzed via the $2^{{‐\Delta \Delta C}_{t}}$ method and expressed as fold change compared with GAPDH. The primer sequences are as follows:

ACRBP: Forward, 5′‐CAGTGACAGAACGCCAGACCTTC‐3′,

Reverse, 5′‐CCTTGCTCCTGCTTGTGCTCTG‐3.

GAPDH: Forward, 5′‐GCACCGTCAAGGCTGAGAAC‐3′,

Reverse, 5′‐TGGTGAAGACGCCAGTGGA‐3′.

### 2.5 Immunohistochemistry (IHC)

ACRBP was detected in OC tissues and normal ovary tissues via immunohistochemical staining. Tissue sections were deparaffinized, rehydrated, and heated in an antigen retrieval buffer (10 mmol/L citrate buffer, pH = 6.0) for 15 min. After blocking endogenous peroxidase activity with 0.3% H_2_O_2_ in phosphate‐buffered saline, rabbit polyclonal antibody against ACRBP (Cat. No. ab64809, Abcam) at 1:200 dilution was added and incubated overnight at 4°C in a humid chamber. Subsequently, horseradish peroxidase (HRP)‐conjugated goat anti‐rabbit IgG (Long Island Biotech, China) was then incubated for 30 min at room temperature, followed by detection system by using 3,3′‐diaminobenzidine (Maixin Biote) and counterstained with hematoxylin. The tissue sections were immunohistochemically stained in strict accordance with the instructions. Brownish yellow or brown particles appeared in the cytoplasm or nucleus as positive cells. The staining intensity of the positive cells and the proportion of positive cells in the total cells were combined to determine the results. The percentage of positive cells showing moderate to strong staining intensity was scored. Score is explained as follows: (−), <5%; (+), 5%–25%; (++), 26%–50%; (+++), >50%. Rabbit IgG monoclonal antibody (bs‐0295P) was used as the isotype control. For statistical analysis, Ki67 index was classified into negative (<10%) and positive (≥10%) categories according to the expression rate of Ki67 in postoperative pathological results.

### 2.6 Enzyme‐linked immunosorbent assay (ELISA)

Antibodies against ACRBP, ACRBP antigens in the serum samples, and the levels of ACRBP in cell‐cultured medium were determined using the Human ACRBP‐Ab ELISA Kit and Human ACRBP ELISA Kit (MLBio) according to the manufacturer's protocol, respectively. In brief, the ELISA kit included serial diluted standards (0, 10, 20, 40, 80, 160 ng/ml) that were used to determine ACRBP serum levels. Serum samples were diluted at a ratio of 1:5, and 50 µl of the samples were pipetted into each well of ELISA plates. Afterward, 100 μl of HRP‐conjugated reagent was added to each well (except blank well) and incubated for 60 min at 37°C. The plates were washed with phosphate‐buffered saline with Tween 20 (PBST) five times. Chromogen solution was then added to each well in the dark and incubated for 15 min at 37°C. Finally, a stopping solution was added, and optical density (OD) values were immediately read at 450 nm by using a microplate reader (Bio‐Rad). Each sample was tested in a duplicate well. The cutoff value for determining a positive reaction was designated as the mean OD value plus two standard deviations (SD; mean + 2 SD) of the control groups.

### 2.7 Statistical analysis

All data management and analyses were performed using the Statistical Program for Social Sciences software 22.0 (SPSS, IBM) and GraphPad Prism 8.0 software. Quantitative variables were expressed as the mean ± SD and analyzed by one‐way analysis of variance (ANOVA) or Student's *t* test. Qualitative variables were compared using Pearson *χ* ^2^‐test or Fisher's exact test. Receiver operating characteristic (ROC) curve analysis was performed by MedCalc v19.0.4. OS and DFS were calculated via the Kaplan–Meier method. Differences in survival curves were compared via the log‐rank test. Results were considered statistically significant at *p < .05 and **p < .01.

## 3 RESULTS

### 3.1 ACRBP expression and prognosis in OC analyzed by database

**Figure 1:**
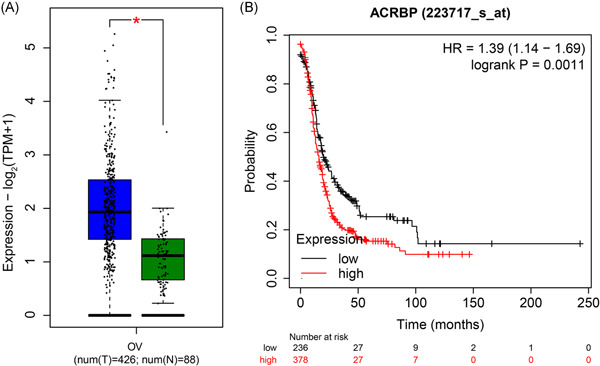
Expression and prognosis of acrosin binding protein (ACRBP) in database. (A) Expression of ACRBP mRNA in ovarian cancer (OC) and normal ovarian tissues using GEPIA database. (B) Survival analysis of ACRBP in OC patients using the Kaplan–Meier plotter database

We used the gene expression data of 426 OC and 88 normal ovarian tissues from the GEPIA database to analyze differences in ACRBP expression between OC and normal ovarian tissues. Results showed that ACRBP mRNA expression in OC tissues was significantly higher than that in normal ovarian tissues (Figure 1A). Survival analysis for ACRBP was performed using the Kaplan–Meier plotter according to the cancer genome atlas (TCGA) data. As shown in Figure 1B, the prognosis of patients with OC was closely related to ACRBP expression. The median survival of the cohort with low ACRBP expression was 20 months, whereas the cohort with high ACRBP expression was 16 months. Patients with high ACRBP expression had poorer OS than those with low expression (*p* < .05), suggesting that ACRBP expression is related to poor prognosis of patients with OC.

### 3.2 ACRBP mRNA and protein detected by clinical OC samples

**Figure 2:**
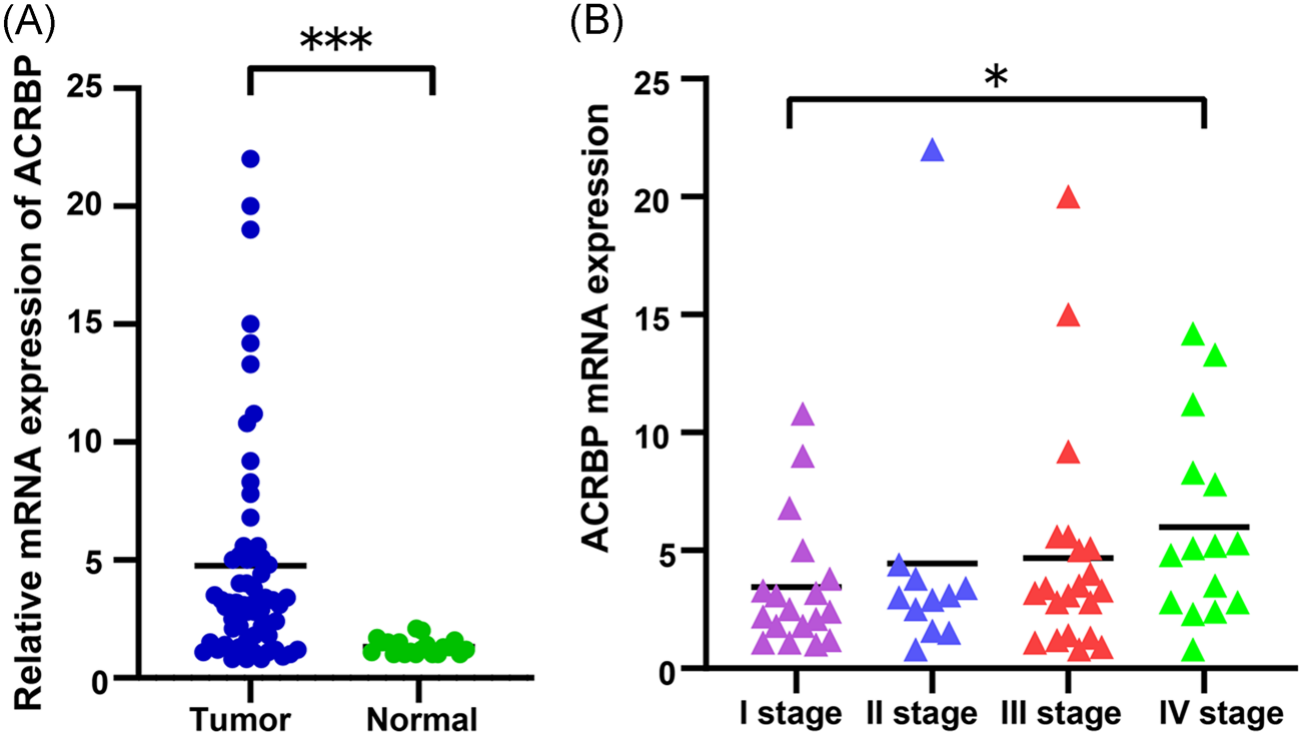
Quantitative real‐time polymerase chain reaction analysis of acrosin binding protein (ACRBP) mRNA expression in ovarian cancer (OC). (A) Expression of ACRBP mRNA in OC and normal ovarian tissues. (B) Expression of ACRBP mRNA in different stages of OC

The clinical importance of ACRBP in OC was further elucidated by evaluating the expression of ACRBP mRNA and protein via qRT‐PCR and IHC, respectively. A total of 65 OC specimens and 20 normal ovarian specimens were collected. As shown in Figure 2A, the ACRBP mRNA level in OC tissues was evidently higher than that in normal ovarian tissues (*F* = 5.3, *p* < .001). ACRBP mRNA expression level in different OC stages was significantly different (Figure 2B).

**Figure 3:**
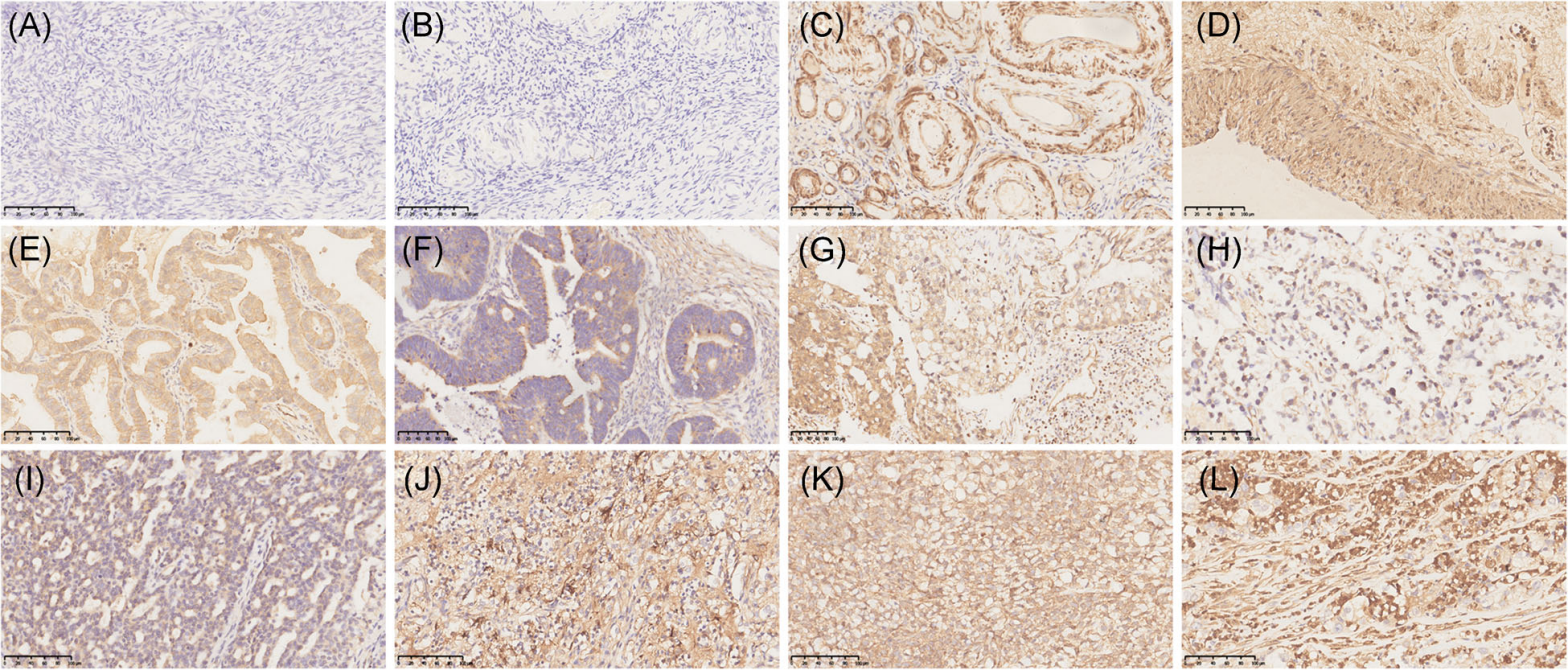
Acrosin binding protein expression pattern in ovarian cancer and normal ovarian tissues. (A and B) Normal ovaries. (C and D) Serous ovarian adenocarcinoma. (E and F) Mucinous ovarian adenocarcinoma. (G and H) Ovarian clear cell carcinoma. (I) Endometrioid ovarian adenocarcinoma. (J) Ovarian immature teratoma. (K) Ovarian granulosa cell tumors. (L) Ovarian malignant Brenner tumor. Scale bar = 100 μm

In this study, we included different pathological types of ovarian malignancies (EOC, ovarian germ cell tumor, ovarian sex cord‐stromal tumor, and other types) and immunohistochemically stained ACRBPs in OC and normal ovarian tissues. A representative result of ACRBP immunohistochemical staining is shown in Figure 3. Results showed that the intensity and quantity of ACRBP expression in OC tissues were higher than those in normal ovarian tissues. ACRBP was mainly expressed in the cytoplasm of OC cancer cells and a patchy staining pattern existed. No ACRBP was detected in the normal ovarian tissues. By contrast, tumor cells showed prominent ACRBP expression, especially in the glandular region. Immunohistochemical staining revealed that ACRBP was highly expressed in all major histological types of OC; 70.7% (46/65) of OC samples presented high ACRBP expression (++/+++) (*p* < .05). Of the samples tested, EOC had a remarkably higher ACRBP expression rate (67.3% [35/52]) than other types of ovarian malignancies (53.8% [7/13]). Furthermore, 80% and 20% of the samples with high (≥10%) and low (<10%) Ki67 expression were found, respectively, on the basis of the postoperative pathology record. No nonspecific staining was obtained when the sections were incubated with preimmune serum as negative control (data not shown).

### 3.3 Correlation between ACRBP expression and clinicopathological features

The effects of ACRBP on OC progression were investigated by analyzing the correlation between ACRBP expression and the clinicopathological parameters of patients with OC, including age, FIGO stage, tumor grade, histopathology, serum CA125 level, lymph node metastasis, and chemosensitivity (Table 1). We divided 65 patients with tumor into high‐ and low‐expression groups with the median (P_50 _= 3.2) of relative mRNA expression as the cut‐off point. Moreover, we classified 65 OC samples into high‐ (++/+++) and low‐expression (−/+) groups on the basis of the ACRBP immunostaining criterion as described in Section Methods, 2. Results showed that both ACRBP mRNA and protein expression levels were closely associated with FIGO stage. Patients with high OC stages (III and IV) showed a higher ACRBP expression level than those with low OC stage (I and II). Furthermore, the difference in the status of ACRBP expression and chemosensitivity was statistically significant (*p* = .02). No correlation was observed between ACRBP expression and other clinicopathological parameters. These results indicated that ACRBP overexpression was strongly associated with the poor phenotype and tumor progression of OC. Patients with higher ACRBP expression tended to have poorer clinical outcome, especially those in the advanced stages and with chemotherapy resistance, than those with lower ACRBP expression.

### 3.4 Presence of ACRBP antibody/protein with clinical importance in the sera of patients with OC

**Figure 4:**
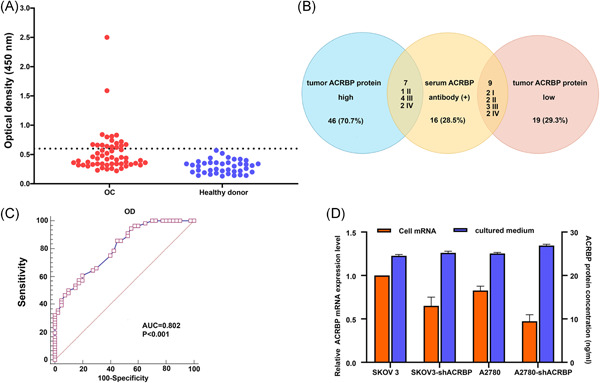
Measurement of antibody against acrosin binding protein (ACRBP) in sera and ACRBP in cultured medium using an enzyme‐linked immunosorbent assay. (A) Detection of ACRBP antibody in the sera from ovarian cancer (OC) patients and healthy donors. (B) ACRBP expression in OC patients with serum antibody positive. (C) Receiver operating characteristic curve of serum anti‑ACRBP antibody in distinguishing OC with normal individuals. (D) Detection of ACRBP mRNA in OC cells and ACRBP in cultured medium of OC cell culture

Given that OC tissues specifically express ACRBP, we investigated whether spontaneous humoral response against ACRBP and ACRBP may occur in the sera of patients with OC. Therefore, we tested the sera from 56 patients with OC and 40 healthy volunteers via ELISA. When the cutoff was set at a value of mean OD + 2 SDs (cutoff = 0.6), antibody against ACRBP was demonstrable in 28.5% (16/56) of the serum samples of patients with OC, but not in the serum samples of healthy volunteers (Figure 4A). Of 16 antibody‐positive sera, there were 7 (7/46, 15.2%) patients with an ACRBP high expression tumor, and 9 (9/19, 47.3%) patients with an ACRBP low expression tumor. And those sera antibody‐positive patients with ACRBP high expression tumor include one in Stage II and six in Stages III and IV, whereas sera antibody‐positive patients with ACRBP low expression tumor include four in Stage I–II and five in Stages III–IV (Figure 4B). No association was observed between serum ACRBP antibody and the clinicopathological parameters of patients with OC (Table 2). In addition, ACRBP was also detected in 7.1% (4/56) of the serum samples of patients with Stages III and IV OC burdened with ACRBP high expression tumor. ACRBP was not detected in the sera of healthy volunteers.

**Table 2:** Correlation between serum ACRBP antibody and clinicopathological characteristics in OC

	Serum ACRBP antibody (*n* = 56)		
Item	Positive/total (%)	*χ* ^2^	*p*
Age (years)			
<50	9/25 (36.0)	0.652	.419
≥ 50	7/31 (22.6)		
FIGO stage			
I–II	5/26 (19.2)	1.308	.253
III–IV	11/30 (36.7)		
Grade			
G3	6/23 (13.0)	0.002	.966
G1 + G2	10/33 (30.3)		
Histopathology			
Serous	7/24 (29.2)		
Mucious	4/13 (30.8)		
Endometriod	2/6 (33.3)	1.312	.859
Clear cell	0/3 (0)		
Others	3/10 (30.0)		
Serum CA125 (U/ml)			
<500	10/27 (37.0)	1.117	.290
≥500	6/29 (20.7)		
Lymph node metastasis			
Positive	7/21 (33.3)	0.093	.760
Negative	9/35 (25.7)		
Chemosensitivity			
Sensitive	12/36 (33.3)	0.562	.453
Resistant	4/20 (20.0)		

Abbreviations: FIGO, International Federation of Gynecology and Obstetrics; OC, ovarian cancer.

Given that ACRBP antibody was present at certain proportions in the sera of patients with OC, we further evaluated whether serum ACRBP antibody can be used as a potential diagnostic marker for OC. ROC curve analysis was performed. The area under ROC curve for prediction of OC by serum ACRBP antibody was 0.802 (95% CI = 0.708–0.876, *p* < 0.001) (Figure 4C). The sensitivity and specificity for this marker were 85.71% and 55.0%, respectively. This result suggested that serum ACRBP antibody may be a moderate serum marker in OC.

### 3.5 Detection of ACRBP in cultured medium of OC cells

Because ACRBP was detected in the sera of patients with OC, we explored whether the ACRBP is secreted by OC cells. SKOV3 and A2780 cells were stably transfected with ACRBP shRNA (sh‐ACRBP). The ACRBP mRNA of these cells was confirmed via qRT‐PCR, and the ACRBP in the cultured medium of the OC cells was detected via ELISA. As shown in Figure 4D, although ACRBP mRNA decreased in the OC cells after downregulating ACRBP, the ACRBP level of cultured medium in the OC cells with or without sh‐ACRBP treatment did not substantially change (range: 24.31–27.20 ng/ml), implying that the ACRBP may not be a secreted protein.

### 3.6 Relationship of ACRBP in tissues and humoral immunity and survival in OC

**Figure 5:**
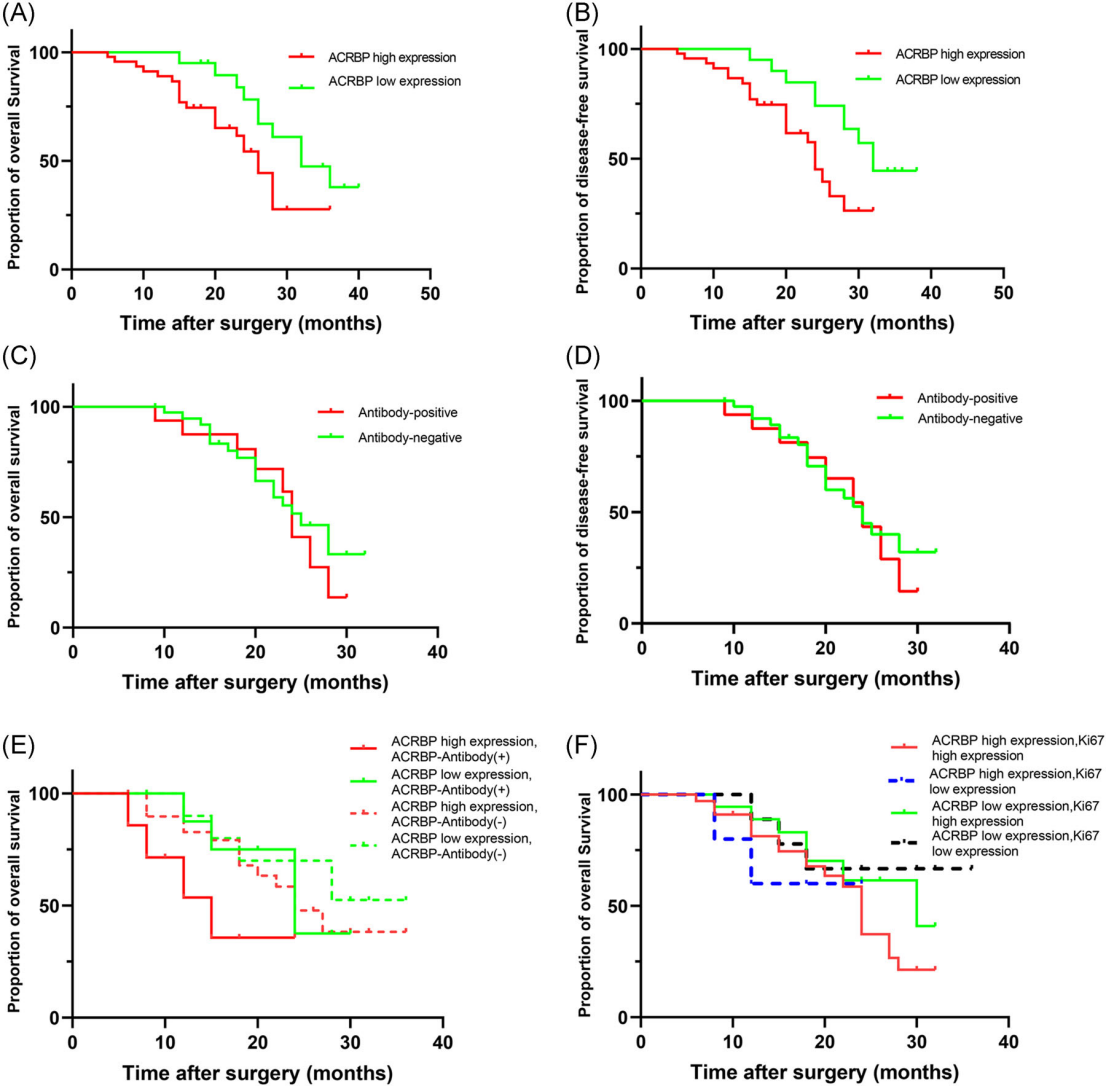
Correlation between acrosin binding protein (ACRBP) protein expression and prognosis of ovarian cancer (OC) patients. (A and B) Kaplan–Meier survival curves of overall survival (OS) (A) and disease‐free survival (DFS) (B) according to ACRBP expression in OC patients. (C and D) Kaplan–Meier survival curves of OS (C) and DFS (D) according to antibody‐positive and antibody‐negative in patients. (E) Kaplan–Meier survival curves of patients with OC displaying different combinations of ACRBP expressions and ACRBP‐antibody. (F) Kaplan–Meier survival curves of patients with OC displaying different combinations of Ki67 and ACRBP expressions

We assessed the association of ACRBP expression in OC tissues and serum ACRBP antibody and survival of patients with OC. In this study, the mean follow‐up time of the 65 patients was 25.2 months (range: 5–40 months). The 3‐year OS and DFS of the patients with high ACRBP expression were significantly lower than those with low ACRBP expression (*p* = .040, *p* = .021) (Figure 5A,B). The median survival time of the patients with high ACRBP expression was 26 months, whereas the median survival of patients with low ACRBP expression was 32 months. Results demonstrated that high ACRBP expression was significantly associated with a poor OS and DFS in OC. However, the difference in OS and DFS between patients with serum antibody positive and serum antibody‐negative was not significant (*p* = .56, *p* = .72) (Figure 5C,D).

We further combined ACRBP in tissues with ACRBP antibody to analyze the 3‐year OS of the patients with OC. The patients were divided into four groups: high ACRBP expression and serum antibody‐positive (*n* = 7), low ACRBP expression and antibody‐positive (*n* = 9), high ACRBP expression and antibody‐negative (*n* = 30), and low ACRBP expression and antibody‐negative (*n* = 10). Survival analysis revealed no significant difference between the four groups (*p* = .27) (Figure 5E). Finally, given that Ki67 is a commonly used tumor marker in clinical settings, we combined the data of ACRBP and Ki67 in tissue sections to analyze their relationship with the OS of patients with OC. The relationship between ACRBP and Ki67 is illustrated in Table 1. Combined ACRBP and Ki67 expression did not show a statistically significant difference in terms of OS (*p* = .34) (Figure 5F). Taken together, these findings suggested that patients with high ACRBP expression had poor prognoses.

## 4 DISCUSSION

Despite treatment with cytoreductive surgery followed by chemotherapy, the clinical outcome of patients with OC remains poor. In recent years, CT antigens or CT genes have attracted increased attention owing to their restricted expression patterns and immunogenicity.^19^ The antigenicity of CTA can be utilized as a target for the design of anticancer vaccines.^20^ Thus, identification and characterization of the CT antigen are of great value in immunotherapy and prognosis monitoring for OC. Given that OC is one of the cancers with a high frequent expression of CT antigens, many patients would benefit from CT antigen‐based immunotherapy even if first‐ and second‐line therapies fail. In this study, we focused on ACRBP, one of the CT antigens, to evaluate its expression profile and serological status with clinical importance in patients with OC.

We analyzed ACRBP expression in database and found that ACRBP was elevated in OC, high ACRBP expression was negatively correlated with the OS of patients with OC. We then detected the mRNA and protein expression levels of ACRBP in our clinical samples of OC. Our results indicated that the relative mRNA expression of ACRBP in OC tissues was 5.3‐fold higher than that in normal ovarian tissues. Tammela et al.^18^ have found that ACRBP mRNA expression in 23% (23/100) of OC specimens. Another study reported by Ono et al.,^12^ ACRBP mRNA was detected in multiple malignancies including bladder cancer, breast cancers, liver cancers, lung cancers, and colon cancers, its frequency was a range from 40% to 15%, but no information was available to OC. Although the results of sensitive qRT‐PCR used herein cannot be compared with those of previous studies that employed conventional RT‐PCR, the proportion of ACRBP mRNA was definitely in OC as well as other tumors. With regard to the expression of the ACRBP, 70% (46/65) of the OC specimens had high ACRBP expression, and this result was observed in all major histological types of OC. In a previous study, we detected the ACRBP in a commercial tissue microarray of OC that demonstrates high expression of the ACRBP (81%, 87/107).^21^ Tammela et al.^18^ also reported high frequency of ACRBP expression in OC tissues, instead of a panel of normal tissues (brain, heart, lungs, skeletal muscles, kidneys, ovary, and stomach). In addition, ACRBP existed in many other tumors such as hepatocellular carcinoma, colorectal cancer, and glioma according to previous studies. These results suggested the utility of ACRBP as a potential marker for OC at the very least.

Our results demonstrated that both ACRBP mRNA and protein expression levels were correlated with the FIGO stage. Moreover, the patients with advanced OC stages showed higher ACRBP expression than those with low OC stages. Furthermore, we found that chemotherapy resistance was related to high expression of the ACRBP but not to mRNA expression, a result that may be attributed to different sampling areas reflecting the heterogeneity of the inside of tumors. Drug resistance is a key factor determining patient prognosis in OC. Few studies investigated this issue with ACRBP. Whitehurst et al.^22^ established that reduced ACRBP expression in OC cells increases the sensitivity to paclitaxel‐induced mitotic defects. Moreover, they observed that ACRBP interacts with NuMA, a mitotic spindle protein. High expression levels of ACRBP and NuMA endow OC cells with aggressive features. ACRBP overexpression may promote chemoresistance and poor survival in EOC. Hu et al.^23^ have proposed that NANOG may be a candidate protein interacting with ACRBP in liver cancer. NANOG has been found to regulate cancer stem cells (CSCs) inside the cancer cells, which show a peculiar potential in tumor progression, heterogeneity, metastasis, recurrence, and drug resistance. NANOG also involved in OC tumorigenesis and chemoresistance.^24^ Therefore, according to the present study and previous works, ACRBP may act as a marker of chemotherapy resistance in OC.

Cancers are immunogenic. Cancer cells aberrantly express antigenic proteins that are not expressed in normal cells. These antigenic proteins induce cancer immunity.^25^ An increasing number of tumor‐associated antigens are being identified, including CT antigens that are widely and highly expressed in various tumors. Humoral responses to CTAs have been found in several cancers.^26^, ^27^, ^28^ Thus, serological surveys for these antigens are badly needed because they may hold clinical value for the diagnosis or monitoring of tumor progression and response to treatment, including chemotherapy and immunotherapy. In the present study, 28.5% (16/56) of the serum samples of patients with OC had the ACRBP antibody, but this antibody was not detected in the serum samples of healthy donors. Moreover, majority of the ACRBP antibody‐positive patients (68.75%, 11/16) were in Stages III and IV. Initially, we had expected that all patients with the ACRBP antibody in their serum samples would burden tumors expressing the ACRBP. However, of the 16 antibody‐positive patients, only 7 (7/46, 15.2%) had tumors with high ACRBP expression, and the rest of them had tumors without or with low ACRBP expression. A plausible explanation for this result is that the presence of serum antibody in patients with negative or low ACRBP expression may contribute to the inherently heterogeneous nature of OC that leads to uneven distribution of the ACRBP in different sampling areas. Moreover, the ability of a patient to produce an immune response against antigen is different, or the ACRBP is very immunogenic in patients with OC; even low levels of the ACRBP can raise and maintain the humoral response for a long time. To date, there was only one study that analyzed 21 serum samples surveyed the presence of the ACRBP antibody in OC. Results showed that 10% of the patients demonstrated the ACRBP antibody in the serum samples. These patients had high expression of the ACRBP as 3+ according to the results of immunohistochemical staining.^18^ By comparison, all patients without ACRBP‐expressing tumors were negative according to the results of ELISA. This discrepancy was most likely due to variations in the samples. Okumura et al.^29^ have identified an HLA‐A24‐restricted ACRBP CD 8 T‐cell epitope, TES_401‐409_, which can be recognized by CD 8^+ ^T cells and induced cytotoxicity against tumor cell line expressing ACRBP mRNA. These findings imply that ACRBP had a capacity to incite a humoral immune response in cancer patients. The level of ACRBP expression might be predictive of humoral immune response and presented in OC patients.

To the best of our knowledge, no report exists in the literature on the ACRBP in the sera of patients with any type of malignancy or disease. In this study, we found four patients with OC with the ACRBP in their sera. ACRBP in the patients' serum was detected at low percentage levels, perhaps we can use ACRBP produced by cells to create a titration curve, find out the detection threshold of our protein, and compare it to the levels detected in serum. This may be a way to improve the positive detection rate of ACRBP in serum. Interestingly, these patients had advanced OC stages and burdened tumors with a high expression of the ACRBP. The reason the ACRBP presented in those patients' sera is difficult to explain. We speculated that the ACRBP entered the blood through the release of necrosed tumor or via secretion by tumor cells. The latter seems less likely because we detected a decrease of intracellular ACRBP expression after shRNA‐ACRBP transfection, but the ACRBP level in the culture medium did not change accordingly. A trace of ACRBP in culture medium could still be detected by ELISA, which was likely because of the fact that tumor cells might have unavoidable necrosis during excessive proliferation, ACRBP in the cytoplasm was passively released from damaged or necrotic cells into the culture medium. This, of course, did not completely rule out that ACRBP was a secretory protein. Therefore, it is necessary to further verify through subsequent experiments. The mechanisms by which ACRBP is produced within cancer cells and enters the circulation must be further investigated.

Given that the ACRBP and antibody were specifically present in OC tissues and the sera of the patients with OC, we further analyzed their relationship with survival. The data demonstrated that high expression of ACRBP in OC tissues was significantly correlated with poor prognosis, whereas the presence of serum antibody against ACRBP did not affect the patients' outcome. When serum ACRBP antibody was combined with protein expression and analyzed, the difference between them in terms of survival rate was not remarkable. In clinical pathology, Ki67 is commonly used to estimate tumor proliferative status. Thus, we also combined the ACRBP with the Ki67 protein to analyze the patients' survival. However, the difference between them was not statistically significant probably because of the relatively small sample size of this study and the insufficient follow‐up time. Thus, more samples must be enrolled to confirm the prognostic value of the ACRBP antibody in OC.

In conclusion, our findings demonstrated that ACRBP expression was markedly upregulated in OC, and its expression was closely associated with the FIGO stage and chemoresistance of OC. Patients with high ACRBP expression tended to have a worse prognosis than those with a low ACRBP expression. ACRBP also displayed inherent immunogenicity. Hence, ACRBP, similar to the other members of CT antigens, could be a potential target for tumor‐specific antigen‐based immunotherapy for patients with OC.

## CONFLICT OF INTERESTS

The authors declare that there are no conflict of interests.

## AUTHOR CONTRIBUTIONS

*Contributed to the conception of the study, analysis, and interpretation of data, and drafted the manuscript*: Lina Lin. *Acquisition of data*: Weixia Nong and Feng Li. *Performed the experiment, validation*: Xia Zeng and Yingying Ge. Helped perform the analysis with constructive discussions: Bin Luo, Rong Fan, and Qingmei Zhang. C*ontributed significantly to analysis and manuscript preparation, and given final approval of the version to be published*: Xiaoxun Xie.

## Data Availability

The data that support the findings of this study are available from the corresponding author upon reasonable request.
